# Comparison of the accuracy of intraoral digital impression system and conventional impression techniques for multiple implants in the full-arch edentulous mandible

**DOI:** 10.4317/jced.57926

**Published:** 2021-05-01

**Authors:** Firas-Abdulameer Farhan, Ali-Jameel-Abdul Sahib, Abdalbseet-Ahmad Fatalla

**Affiliations:** 1 PhD. Senior Lecturer, Department of Prosthodontic, College of Dentistry, University of Baghdad, Bab Al-Muadham campus of the University of Baghdad, 1417, Baghdad, Iraq; 2 MSc. Assistant professor, Department of Prosthodontic, College of Dentistry, University of Baghdad, Bab Al-Muadham campus of the University of Baghdad, 1417, Baghdad, Iraq; 3 PhD. Professor, Department of Prosthodontic, College of Dentistry, University of Baghdad, Bab Al-Muadham campus of the University of Baghdad, 1417, Baghdad, Iraq

## Abstract

**Background:**

Several impression techniques, especially in combination with computer-aided design and computer-aided manufacturing (CAD/CAM), are used in increasing the accuracy of dental implantology and decreasing patient discomfort. The study was designed to examine the accuracy of the digital impression (DI) of multiple implants with an intraoral scanner (IOS) and compared with that of a conventional impression (CI).

**Material and Methods:**

Four dental implants were placed in teeth area #33, #36, #43 and 46# of the mandibular full-arch model. The implanted model was replicated by IOS and CI after fitting of scannable abutments over the implant screws. Then, a small hole was made on the scan region (as a reference point). Two types of CI techniques were used; dual-phase (DP) and monophase (MP). Stone casts were produced through a conventional close tray impression technique using die stone. The casts were scanned with a laboratory scanner. DI was attained by scanning the implanted model with the IOS. The control sample was accomplished by scanning the implanted model directly with a laboratory scanner. Dimensional accuracy was calculated by measuring the distances between the reference points of four measuring parameters as follows; A-B, B-C, C-D, and A-D using CAD software.

**Results:**

The mean values and standard deviation between the four parameters of different impression techniques (CI and DI) and the control group showed convergent value. One-way ANOVA test showed all CI techniques, except IOS, showed a significant differences from the control group.

**Conclusions:**

Compared with CI, the IOS was more accurate because no differences were observed between its measurements and those of the control model. CI is simple and reduces patient discomfort when used in fabricating multiple implants and allowing communication with dental technicians.

** Key words:**Dimension accuracy, conventional impressions, digital impressions, multiple implants.

## Introduction

Dental implants have become a successful restorative treatment modality in modern clinical dentistry. Despite that surgical and restorative phases have complexities, technological advances in computer tomography (CT), guided surgery, and diagnostic tools have rendered the surgical aspect increasingly predicTable and less complex (1).

One of the reasons for the unfamiliarity and lack of use of implants in dentistry is the perceived difficulty in making implant impressions. Dental impressions are a critical step in implant dentistry. The imprecise transfer of an implant location can lead to an ill-fitting prosthesis, which may ultimately result in both biological and mechanical difficulties (2).

Currently, the advancement of computer-aided design and computer-aided manufacturing (CAD/CAM) has enabled the generation of digital impression from the patient’s mouth through digitizing a conventional impression (CI) with a laboratory scanner (3). CI is used in fixed prosthodontics supported by dental implants for a long period. However, this kind of impression has several limitations, including the selection of tray and type of impression material, impression technique, time consumption, impression disinfection, and casting material used to produce a cast model and difficulty of cast model storage (4) These limitations prompted researchers to search alternative impression techniques, such as the digital impression (DI) technique (5) 

Intraoral scanner (IOS) is a device used in capturing direct optical impressions in dentistry (6). Similar to other three-dimensional (3D) scanners, an IOS emits a light source (laser, or more recently, structured light) onto the object with imaging sensors that were processed by the scanning software. An IOS scans a prepared tooth or teeth and implant scannable abutments which are a cylindrical shape and secure by the implant screws. Scannable abutment is fabricated by a variety of materials, such as titanium alloy, polyetheretherketone (PEEK) and various resin. A scannable abutment has an important role in transferring the 3D implant position by IOS (3,7).

The accuracy of the impression is the main factor influencing the assembly’s fit, which is affected by impression material, impression technique, implant angulation, and the number of implants (8,9). An implant-fixed prosthesis should perfectly fit for it to last for a long period. Any improper restoration fitness may lead to the mechanical complications of dental implant, such as screw loosening or fracture (10) and biological problems, which could compromise the bone-implant interfaces and the homogeneity of the occlusal load (11).

Conventional impression (CI) techniques using tray and impression material cannot eliminate the error of expansion, shrinkage, and distortion of impression or gypsum material. An IOS provides a means to overcome or minimize such errors (12).

This in-vitro study aimed to evaluate the accuracy of the full-arch edentulous mandible with four dental implants scanned by an IOS and compared it with two types of CI materials.

The null hypothesis is that different implant impression techniques used in this study have no significant differences.

## Material and Methods

-Preparation of implant model and surgical guide 

A mandibular implant practice model (Dentium, Co., Ltd., Korea) was used in simulating the edentulous site of the patient mouth. Four dental implants were positioned on the teeth area of the edentulous model (#33, #36, #43, and #46) with an implant guide kit (Dentium, Co., Ltd., Korea). The model was scanned with a laboratory scanner of CAD/CAM machine for fabrication of a surgical guide, which was punctured according to previously determined implant sites.

-Installation of dental implant 

Four implant sites were prepared according to the standard instructions used in dental implant procedure. A pilot drill (2.4 mm diameter) was inserted through each entrance point of the surgical guide hole with a depth of 10 mm, followed by two intermediate drills (3.0 and 3.5 mm in diameter) and a final drill (4 mm in diameter). A screw root-form endosseous Ti-6Al4V implants (FXS4010 (D), Dentium, Co., Ltd., Korea) was inserted and tightened using a manual ratchet in the prepared hole according to standard insertion torque recommended by the manufacturer’s instructions.

-Location of scannable abutments

**Figure 1:**
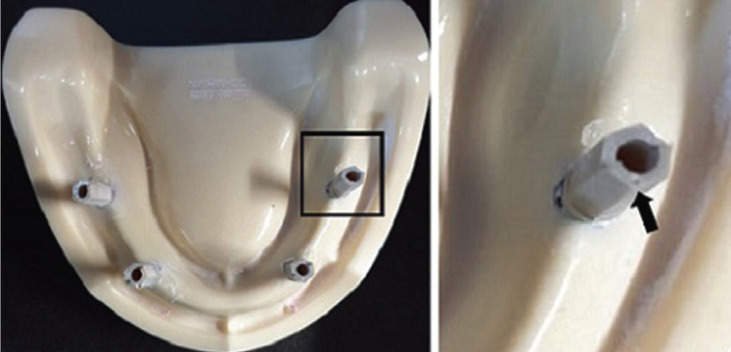
The implanted model received scannable abutments with a small notch (black arrow) as a reference point.

Four scannable abutments were positioned and tightened into the implant analogs using 15 Ncm torque. The scan region of the scannable abutment was specified with a small notch (reference point) positioned at the mesial aspect of scan region of each scannable abutment using a low-speed handpiece and small fine round bur (Fig. 1). The points were used as standard measurement parameters of accuracy for different impression techniques. Two types of impression technique were proposed, which were divided according to the type of impression system into; CI and DI.

-Preparation of conventional impression technique

The CI technique was subdivided according to the type of impression materials into; dual-phase impression material (heavy and light body) condensation silicon (elite HD, Zhermack clinic, Italy) as a group DP, and monophase vinylpolysiloxane (addition silicone) (Hydrorise, Zhermack clinic, Italy) as a group (MP). A conventional close tray impression technique was used in both DP and MP impression materials. The impressions were poured with type IV dental die stone material (Elite Rock, Zermack, Italy) to produce a master stone model with a scannable abutment. The stone model was scanned with a laboratory scanner (rainbow™ Digital Dentistry, Dentium, Co., Ltd., Korea) for conversion of the stone cast to digital data.

-Preparation of digital impression technique

For the DI technique, Medit-i500 IOS was used (Dentium, Co., Ltd., Korea) and lower than 1000 images were made for the mandibular arch according to the manufacturer’s instructions. The control group was achieved by scanning the implanted model directly with a laboratory scanner (rainbow™ Digital Dentistry, Dentium, Co., Ltd., Korea).

-Evaluation of impressions accuracy 

**Figure 2:**
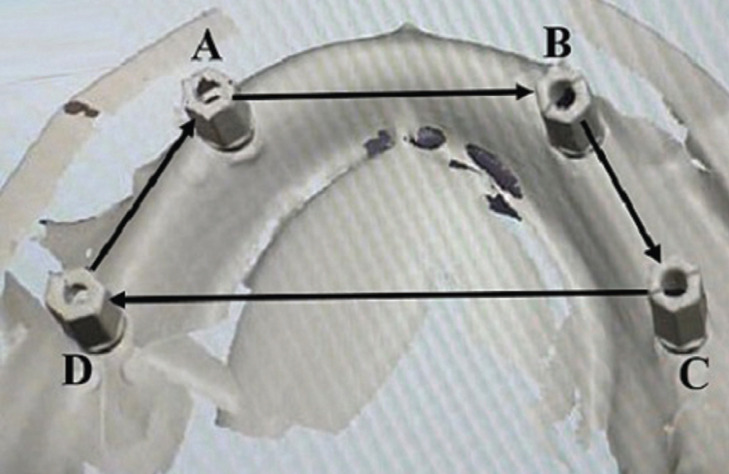
Standard measuring parameters of impression accuracy by software.

The data obtained using the laboratory scanner and IOS were imported into Exocad Dental DB 2.2 Valletta (exocad GmbH, Germany) software for the production of 3D models. The Measuring accuracy of CD and DI techniques were obtained by calculating the linear distance between the reference points of four scan bodies. The reference points were designated as A, B, C, and D which were considered standard parameters used for the measurements. The linear distance measurements were accomplished from A-B, from B-C, from C-D, and D-A respectively as shown in Figure 2.

-Statistical analysis

The calculated impression accuracy was analyzed with one-way analysis of variance (ANOVA) and post hoc multiple comparisons by Bonferroni test between groups at a significance level of *P*<0.05 using Statistical Package for Social Sciences (SPSS v.26.0, SPSS Inc., USA).

## Results

-CI and DI techniques accuracy measurements

The descriptive statistics were presented as mean values and standard deviation (SD) between reference points A to B, A to D, B to C, and C to D of different impression techniques (CI and DI). The values of control group were close to those obtained from the reference points of DI and CI (Table 1).

**Table 1:**
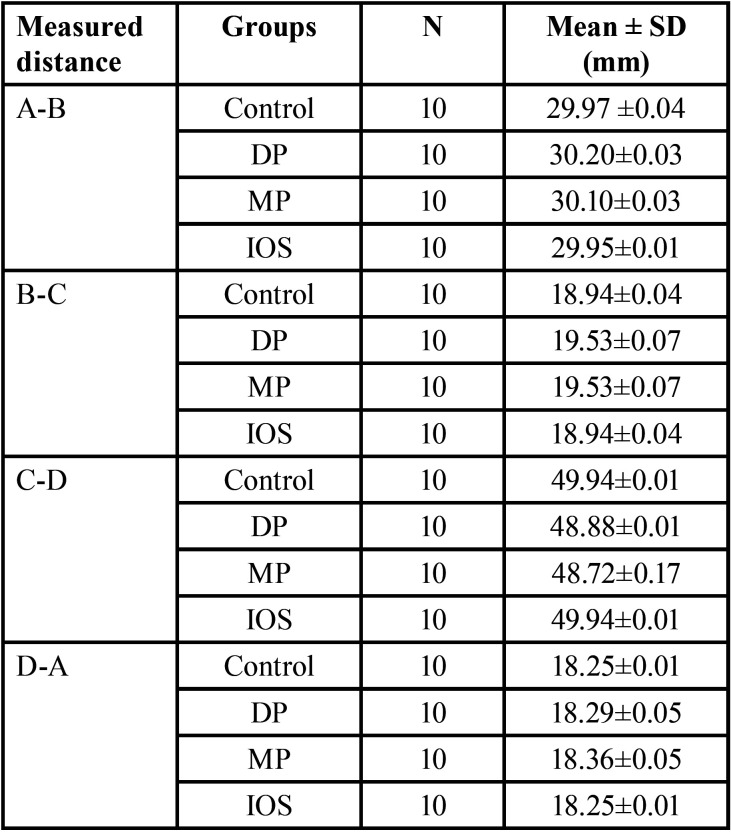
Descriptive analysis between two reference points of different impression techniques (CI and DI groups).

**Figure 3:**
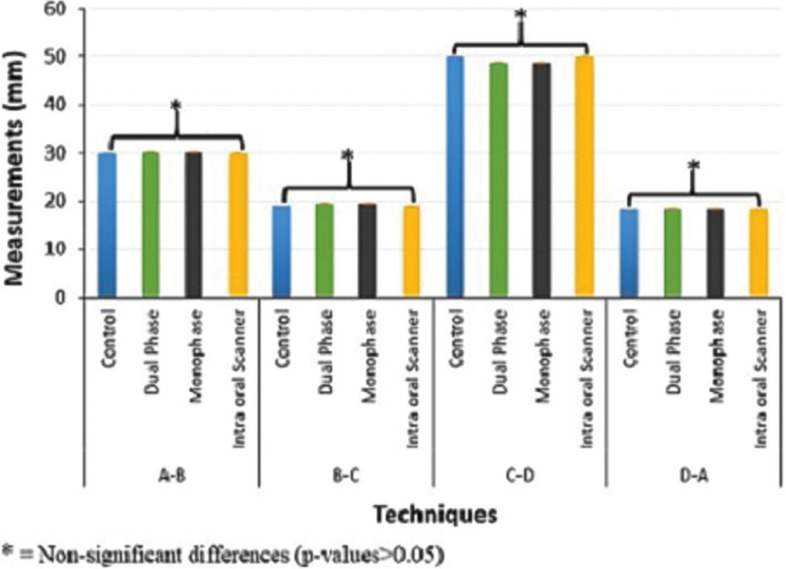
Means and standard deviation between reference points of different impression groups with multiple comparisons among the tested groups.

Multiple comparisons by one-way ANOVA test were conducted and the results showed a significant difference between tested groups, expect IOS group and the control group as illustrated in Table 2 and Figure 3.

**Table 2:**
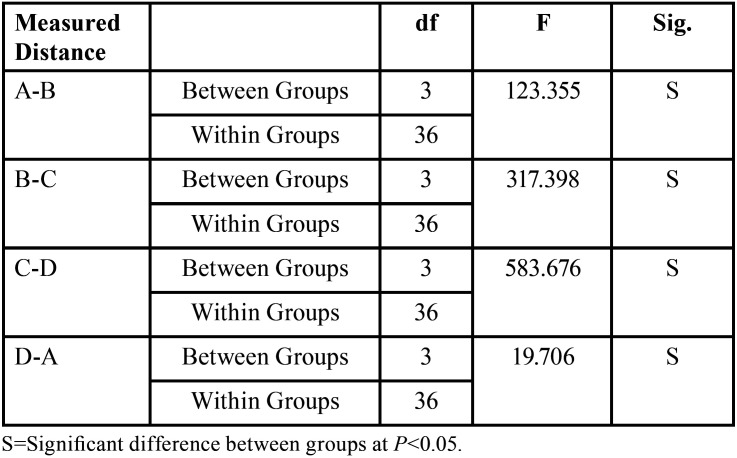
One-way ANOVA test between reference points of different impression techniques (CI and DI groups).

The mean and standard deviation of the overall reading was obtained for each impression technique (Table 3). The highest mean values were found in the control and IOS groups. The One-way ANOVA Table (Table 4) shows that significant differences were found among the groups compared.

**Table 3:**
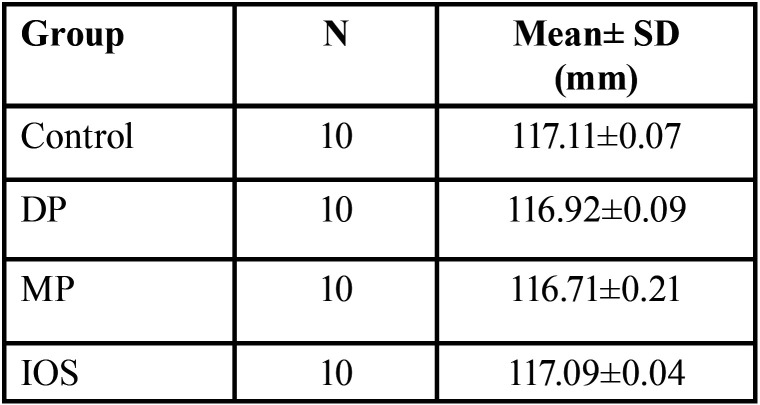
Descriptive analysis of readings summation of linear distances between reference points of each impression technique.

**Table 4:**
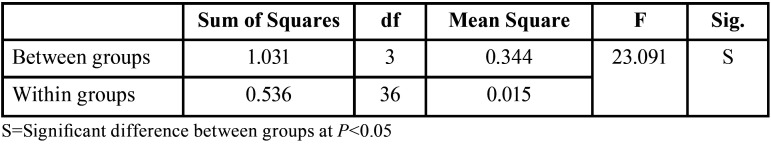
One- way ANOVA test of readings summation for different impression techniques.

**Figure 4:**
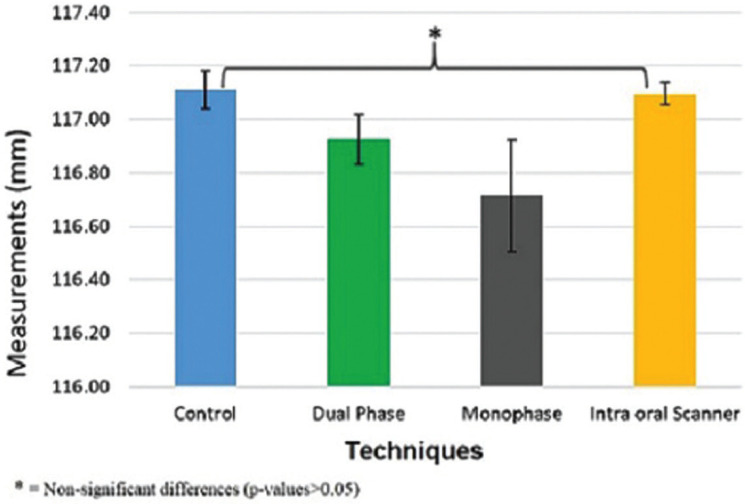
Means and standard deviation of readings summation of linear distances between reference points of each impression technique.

Multiple comparison Bonferroni test was performed on overall mean values measured. No significant difference was found between the control and IOS groups as shown in Figure 4.

## Discussion

The use of IOS has increased in the field of implant dentistry with the development of digital technologies. Studies focusing on edentulous models with multiple implants and demonstrating the accuracy of IOS technologies are few. The present study used a full-arch edentulous model due to increasing number of requests from edentulous patients who want to restore their teeth with fixed prosthesis, which is considered a highly accepTable treatment for a wide range of patients compared with removable complete denture (13,14)

Four implants were placed in selected edentulous mandibular full-arch model sites according to previous studies (15,16). The accuracy of the impressions were evaluated after the conventional impression (stone casts) and implanted model (control group) were converted into 3D models through indirect digitalization (laboratory scanner). A standardized measurement method between the study models of the impression techniques proposed in this study. This method was achieved by using a standard notch (reference point) on the scan body and using CAM software tools to measure the distance between two indexed notches and the standard parameters of each impression technique.

The linear distance differences between A-B, B-C, C-D, and D-A reference points were determined in all impression techniques (Table 1). The null hypothesis should be rejected, as a significant result obtained. Significant differences between control and CI groups were observed, whereas the difference between the control and ISO groups was non-significant (Figs. 3,4). These results showed that the digital impression by IOS is more accurate than the dimensions obtained from the control group with extraoral scanner, consistent with the results presented by many researchers who have examined the dimensional accuracy of digital models produced by IOS and CI technique (17,18). The reports recommended that accuracy is higher in the digital models than that obtained by fabricating definitive casts for extraoral digital impression (CI group) (19,20).

Meanwhile, CI impressions showed significant differences with the control group possibly due to dimensional changes caused by potential laboratory errors, such as shrinkage, irregular thickness or detachment of impression material, and distortion of the impressions (21,22). The reason for the use of intraoral digital impression systems is their satisfactory accuracy compared with that of conventional techniques and extraoral digitization scanner of stone casts, indicated by the conflicting results. The comparison of the results showed that minimal dimensional changes and accurate oral strictures details can be achieved by using the IOS in dental implant treatment (23). Moreover, the digitizing impression data can be documented and manipulated using a CAD/CAM software and used in achieving a high marginal fit and good occlusal adjustment (24,25).

In addition, the use of an IOS will reduces patient discomfort, time-efficient and simplify clinical procedures for dentist and laboratory technician (26,27). Furthermore, IOS decreases the risk of cross-infection by eliminating plaster models and facilitating communication with the dental technician, especially with the presence of the corona virus disease 19 (COVID-19) pandemic, because the main infection pathways of viruses are the air and direct contact (28,29). Moreover, IOS reduces direct contact to oral structure and saliva, which are considered the early target of COVID-19 (30) by reducing the time and minimizing or eliminating the repetition of impression procedure.

## Conclusions

Within this *in vitro* study limitation, the digital scanning systems are superior in accuracy to the conventional impression techniques and can be used in fabricating accurate long-span multiple implant-supported fixed restorations.
